# Recent Developments for Flexible Pressure Sensors: A Review

**DOI:** 10.3390/mi9110580

**Published:** 2018-11-07

**Authors:** Fenlan Xu, Xiuyan Li, Yue Shi, Luhai Li, Wei Wang, Liang He, Ruping Liu

**Affiliations:** 1 School of Printing and Packaging Engineering, Beijing Institute of Graphic Communication, Beijing 102600, China; lvjuan@mail.tsinghua.edu.cn (F.X.); lixiuyan@binn.cas.cn (X.L.); caomeijuan@bigc.edu.cn (Y.S.); liluhai@bigc.edu.cn (L.L.); wangwei@bigc.edu.cn (W.W.); 2 Beijing Engineering Research Center of Printed Electronics, Beijing 102600, China; 3 State Key Laboratory of Advanced Technology for Materials Synthesis and Processing, Wuhan University of Technology, Wuhan 430070, China

**Keywords:** flexible pressure sensor, transduction mechanism, e-skin, wearable sensors, sensibility

## Abstract

Flexible pressure sensors are attracting great interest from researchers and are widely applied in various new electronic equipment because of their distinct characteristics with high flexibility, high sensitivity, and light weight; examples include electronic skin (E-skin) and wearable flexible sensing devices. This review summarizes the research progress of flexible pressure sensors, including three kinds of transduction mechanisms and their respective research developments, and applications in the fields of E-skin and wearable devices. Furthermore, the challenges and development trends of E-skin and wearable flexible sensors are also briefly discussed. Challenges of developing high extensibility, high sensitivity, and flexible multi-function equipment still exist at present. Exploring new sensing mechanisms, seeking new functional materials, and developing novel integration technology of flexible devices will be the key directions in the sensors field in future.

## 1. Introduction

Nowadays, there is rapid development of high-performance smart materials, as well as intelligent home and internet of things (IoT) technology; therefore, sensors technology has gradually stepped into people’s lives and attracted widespread interest from researchers, especially in flexible pressure sensors. The flexible pressure sensors have a wide range of applications because they have excellent mechanical and electrical properties, such as high flexibility, high sensitivity, high resolution ratio, and rapid response, among others [1]. Studies on flexible wearable sensor devices have been rapidly increasing in the last decade.

In the last decade, flexible devices demonstrated great potential in various fields. One of the most important applications is flexible electronic skin (E-skin) for pressure sensing, which is firstly introduced with polymer-based switching matrices for displays, robots, and others [2]. Recently, many review papers focused mainly on the development of flexible electronic devices for E-skin [3,4,5]. Tactile-sensing simulation of the human skin characteristics with high sensitivity, high resolution ratio, and fast response to temperature and pressure is a highly interesting but difficult challenge for applications in health care monitoring and robotic artificial intelligence [6].

**Figure 1:**
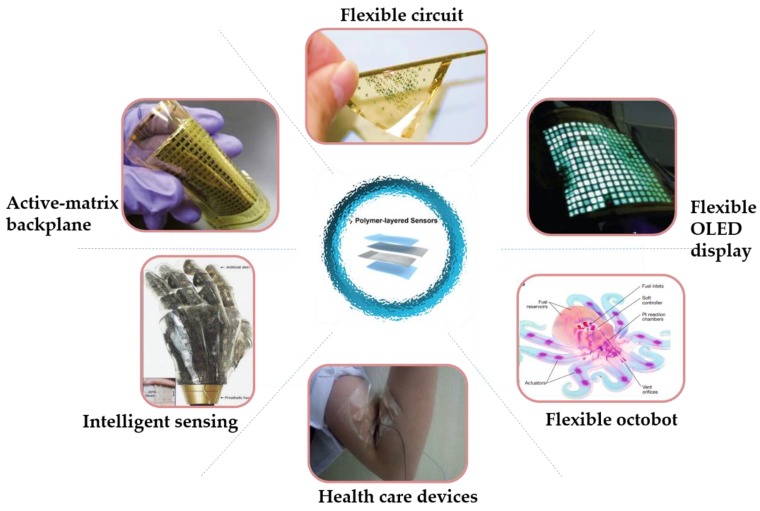
Characteristics and functions or applications of recently developed devices for flexible sensors. “Active-matrix backplane”, reproduced with permission [7]. Copyright 2010, Nature Materials. “Flexible circuit”, reproduced with permission [8]. Copyright 2010, Nature Materials. “Flexible display”, reproduced with permission [9]. Copyright 2013, Nature Materials. “Flexible octobot”, reproduced with permission [10]. Copyright 2016, Nature. “Human motion monitoring”, reproduced with permission [11]. Copyright 2017, ACS Applied Materials & Interfaces. “Intelligent sensing”, reproduced with permission [12]. Copyright 2014, Nature Communications. OLED—organic light-emitting diode.

Takei et al. [7] developed a flexible pressure sensor array (18 × 19 pixels) with macroscale (7 × 7 cm^2^) integration of parallel nanowire (NW) arrays as the active-matrix backplane employed as an artificial E-skin. The miniaturization of sensors and electronic circuits based on microelectronics has played a vital role in the development of wearable devices. The flexible circuit is an important example of this technology. Flexible pressure sensors are important components of E-skin, and the combination of biomedicine and sensing technology for human health monitoring is the research focus of sensors. A flexible, pressure-sensitive, organic thin film transistor with great bending stability, high sensitivity, fast response, high stability, and low power consumption was fabricated by Sekitani [8], demonstrating that the transistors and circuits can be fully integrated into a radius of 100 μm without degrading performance. In addition, Wang et al. [9] proposed a user-interactive E-skin that provides instant visual response through a built-in, active-matrix organic light-emitting diode (OLED) display, and the intensity of the emitted light quantifies the amount of pressure applied. Soft robots represent an emerging field that could achieve better human–computer interaction and adapt to a changeable natural environment, compared with conventional robots composed of rigid materials, such as the octobot [10], and could be widely used in human motion monitoring [11]. Flexible sensors applied in artificial intelligence are still a hot spot of E-skin research nowadays, inspiring the development of artificial skin with multi-modal sensing capability [12], as shown in Figure 1.

## 2. Flexible Pressure Sensors

The development of pressure sensors is traced back to 1954 and based on the discovery of Smith, who explored the compressive resistance effects of silicon and germanium [12,13,14]. Associated with the rapid growth of intelligent robots and wearable electronic equipments [15,16], various new-type devices based on flexible pressure sensors are in high-speed development, for applications in entertainment, games, cars, consumer electronics, industries, and health care fields, among others. The perception mechanisms of flexible pressure sensors can be simply divided into piezoresistivity, capacitance, and piezoelectricity [17,18]. Knowing the performances of these sensors plays a vital role in different application fields so as to reach the best functional matching. Table 1 summarizes some reported results of performances of flexible pressure sensors [19,20,21,22,23,24,25,26].

Studies indicated that some conductive polymeric materials have the effect of piezoresistivity, however, it is difficult to achieve the effect of piezoresistivity for insulating polymeric materials [27]. In recent years, new research result shows that conductive fillers make insulating polymeric materials transform into conductive materials.

Therefore, the choice of materials has always been a core issue that experts are committed to exploring, especially conductive fillers. Gong et al. [28] developed an ultra-thin sensing using a gold film with a thickness of 1.64 μm on a polydimethylsiloxane (PDMS) elastomeric substrate by a casting method, which has 300% strain and fast response (<22 ms). However, the cost of gold film is high, which will pose negative effect on the cost of devices/systems. Generally, the initial conductive fillers belong to a metal-based system. Therefore, researchers are devoted to developing suitable conductive fillers, and the carbon system attracts the attention of researchers owing to its stable electrical performance, low cost, and rich variety; the characteristics of metal and carbon systems are elaborated in Table 2. Doshi and Thostenson [29] fabricated a textile pressure sensor based on carbon nanotubes (CNTs) using fiber/fiber contact and the formation of a sponge-like piezoresistive nanocomposite between fibers that caused changes in electrical conductivity. Such piezoresistive sensors are highly sensitive to touching, and have a high electrical stability. 

### 2.1. Transduction Mechanism

#### 2.1.1. Piezoresistive Pressure Sensors

**Figure 2:**
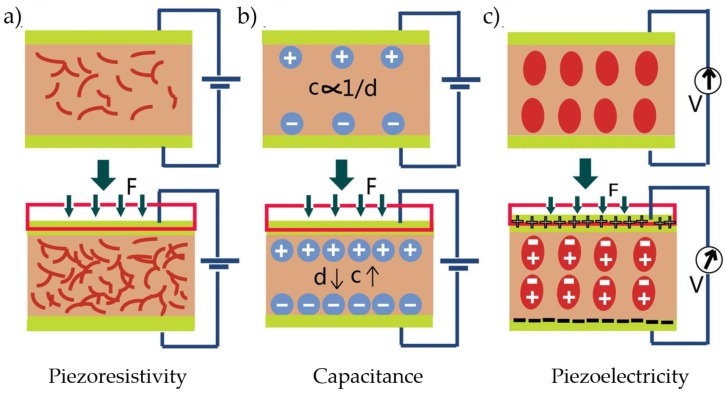
Schematic illustration of three common transduction mechanisms and representative devices: (**a**) piezoresistivity; (**b**) capacitance; and (**c**) piezoelectricity.

The piezoresistive effect-based sensors distort the composite via exerting external force, indirectly changing the distribution and contact status of conductive fillers inside, and then resulting in the resistance of composite changing regularly, which is presented in Figure 2a. Thus, they do not require complex sensor structure, and a low power consumption, wide range of pressure test, and simple manufacturing process cause this kind of sensors to be studied extensively compared with capacitive and piezoelectric pressure sensors. They can be applied in medical examination, sealing inspection, physical exercise, and so on [30,31]. As we know, there are some commercial products in the market, such as the incredible smart bra, which can monitor the heartbeat by resistance variation to prevent the occurrence of diseases [30].

In the past decade, different kinds of sensing materials have been studied to develop high-performance piezoresistive sensors. The studies are mainly focused on fillers with conductive properties. The commonly applied materials are metallic particles; metal NW; and carbon-based materials including carbon black, CNTs, and reduced graphene oxide (rGO) [32].

**Figure 3:**
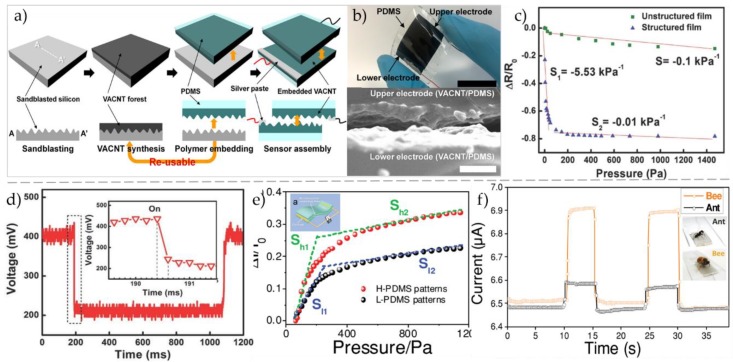
(**a**) Schematic illustration of the fabrication process of the flexible pressure sensor. (**b**) Digital image of the fabricated sensor and its cross-sectional scanning electron microscope (SEM) image. Reproduced with permission [24]. Copyright 2017, ACS Applied Materials & Interfaces. (**c**) Sensors sensitivities with microstructure and no structure. (**d**) Response time of sensors with microstructure. Reproduced with permission [33]. Copyright 2014, Small. (**e**) Sensitivities of pressure sensors constructed with different polydimethylsiloxane (PDMS) microstructures (Inset: schematic of a typical E-skin). (**f**) Real-time I–t curves of the E-skin constructed with PDMS for detection of a bee (40 mg) and an ant (10 mg), respectively. Reproduced with permission [34]. Copyright 2014, Advanced Materials. VANCT—vertically aligned carbon nanotube.

Kim et al. [24] presented a wearable resistance-type pressure sensor with a simple structure and high sensitivity based on a vertically aligned CNT (VACNT) composite conductor embedded in a polymorphic PDMS matrix. Figure 3a shows the schematic illustration of the fabrication process of this flexible pressure sensor and Figure 3b shows the cross-sectional scanning electron microscope (SEM) image of the sensor (scale bar: 10 μm). The sensor delivers a high sensitivity of 0.3 kPa^−1^ and a fast response time of 162 ms, which can be employed to detect joint movements of human body.

In 2014, Chen et al. [33] prepared a “sandwich” structure of a flexible resistance pressure sensor, which was assembled by sealing microstructured electrode and indium-tin oxide (ITO)/polyethylene terephthalate (PET) film. The resistance-type flexible pressure sensor can work under the environmental pressure of <100 Pa, with a relatively high sensitivity of 5.5 kPa^−1^ and lower detection limit of 1.5 Pa, as shown in Figure 3c. Meanwhile, it has a quick response time of 0.2 s, as shown in Figure 3d. Because of its unique stability, the sensor has great potential in the fields of prosthesis and robot artificial intelligence. In the same year, Zhang [34] developed a kind of resistive pressure sensor by transferring microstructure onto silk with PDMS. Figure 3e illustrates the configuration of this flexible E-skin, which is constructed by two layers of single-walled carbon nanotubes (SWCNTs)/PDMS film with the patterned surfaces placed face-to-face. The sensitivity of the sensor increases rapidly when the applied pressure is less than 300 Pa, as shown in Figure 3e. Figure 3f indicates the high sensitivity and fast response time, and a very small pressure could be detected by this sensor, like the weight of an ant or a bee. Furthermore, in order to study the influence of the density of PDMS microstructure on the sensitivity of the sensor, the sensitivities of the E-skin devices based on low-density PDMS (L-PDMS) and high-density PDMS (H-PDMS) are compared. It can be found that the sensor sensitivity of H-PDMS is much higher than that of L-PDMS, that is, about 2.3 times that of L-PDMS. Tran et al. [35] designed a novel piezoresistive micro pressure sensor based on cross-beam film and peninsula (CBMP) diaphragm structure. Compared with other types of sensors, the sensitivity of the sensor is significantly improved. The experimental data showed that the sensor has a pressure range of 0–5 kPa at room temperature and a sensitivity of 25.7 mV/kPa, which is suitable for applications in microelectromechanical systems (MEMS).

#### 2.1.2. Capacitive Pressure Sensors

Capacitive flexible pressure sensor, a device based on the principle of parallel plate capacitor, has the advantages of high sensitivity, fast response, and wide dynamic range. Its working mechanism is changing the capacitance of the sensor by altering the distance between plate capacitors when applying external force, as shown in Figure 2b. The capacitance could be calculated by *C* = *εA*/*d*, where *ε* is the dielectric constant, and *A* and *d* are the area and the distance of two electrodes, respectively [36]. The capacitance is inversely proportional to the distance between plates, and is proportional to the dielectric constant and effective plate area. 

External pressure could change the displacement of elastic materials to respond to the shear force as *A* changes, forward force as *d* changes, and tension as the two parameters change together. For the dielectric layer, it is difficult to achieve high sensitivity of sensors because of a high Young’s modulus of elastomer material, such as some elastomer dielectrics including PDMS with a small Young’s modulus of as low as 5 kPa [37,38].

In recent years, many high-sensitivity capacitive sensors have been reported. Huang [39] designed a new three-axis force sensor consisting of four capacitors based on the change in capacitance caused by triaxial forces. A large number of measurement data showed that the sensitivities of the sensor unit to normal force, x-axis, and y-axis shear force are 0.0095, 0.0053, and 0.0060 N^−1^, respectively, which proved that it has great application potential in the field of skin sensing. Dobrzynska developed a three-axial force sensor based on flexible substrates with finger-shaped electrode capacitors, and measured its capacitance change through applying a three-axial load [40]. The mechanism analyzes the direction and size of three-dimensional (3D) force the sensor received. Besides, they demonstrated the electrical characterization of a typical single sensor in the absence of load to measure cut-off frequency of the device.

**Figure 4:**
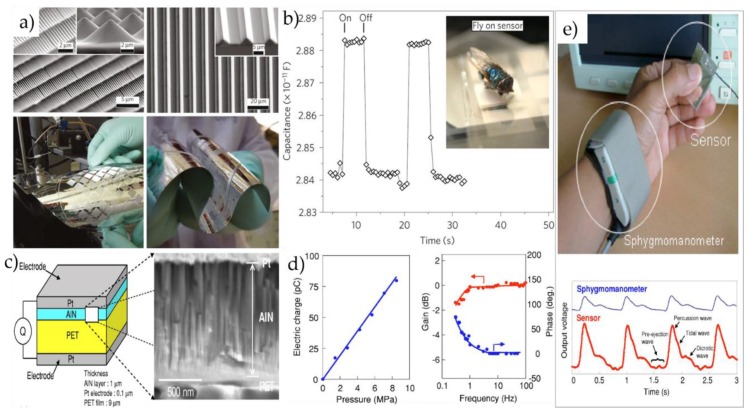
(**a**) (**Top**), SEM images of the microstructured polydimethylsiloxane (PDMS) films. Two-dimensional arrays of square pyramids (**left**) and pyramidal feature arrays (**right**). (**Bottom**), the pressure-sensitive structured PDMS films show the flexible and conformability. (**b**) The microstructured PDMS films are able to sense very small pressure. Reproduced with permission [26]. 2010, Copyright Nature Materials. (**c**) Multilayer structure of flexible pressure sensor, and schematic diagram and SEM image of sensor structure showing Pt electrodes, aluminum nitride (AlN), and polyethylene terephthalate (PET) films. (**d**) Relationship between pressure and electric charge of sensor. The frequency is 10 Hz. Dependence of gain and phase of sensor on frequency. The load and offset are 20 and 40 N, respectively. (**e**) Photograph of actual test setup to measure pulse wave forms with sensor and sphygmomanometer. Human pulse waves measured with sensor and sphygmomanometer. Reproduced with permission [43]. Copyright 2006, Journal of Applied Physics.

Mannsfeld et al. [26] fabricated a flexible capacitive pressure sensor with high sensitivity and instant reaction, which employed the flexible PET/ITO as electrode and silicone rubber with microordered structure as dielectric layer. As shown in Figure 4a,b, Peng et al. [41] developed a kind of capacitive tactile sensing array using PDMS as its structural material. It could detect subtle pressure change by altering the area of capacitance substrate and the distance between substrates to change sensor resolution. This kind of flexible touch sensor could be inserted into human muscle tissues. In addition, Zhuo et al. [42] prepared a micro-capacitive flexible pressure sensor using PDMS as the dielectric layer. The results showed that the sensor with microstructured PDMS layer can achieve high sensitivity, fast response, and strong adaptability to the environment, and can accurately monitor the physiological signals of the human body in real time, such as monitoring the pulse.

#### 2.1.3. Piezoelectric Pressure Sensors

Flexible piezoelectric pressure sensors have received much more attention from researchers because of their facile materials preparation, low cost, easy electrical signal acquisition, and other merits. They are mainly composed of piezoelectric sensitive materials, which can convert mechanical energy and electric energy into each other. Their transduction mechanism could be described as follows: when the material is deformed by external pressure, positive and negative charges separation occurs within the functional material. On the two opposite surfaces of the material, there will appear positive and negative charges arranged in opposite directions, and a potential difference will be formed inside. These potential differences are examined to determine the effect of external forces. As shown in Figure 2c, the features of piezoelectric sensors in detecting high frequency pressure cause them to be applied to the direction of precision equipment manufacturing. Besides, the emergence of new piezoelectric materials, including poly(vinylidenefluoride-trifluoroethylene) (P(VDF-TrFE)), barium titanate (BaTiO_3_), lead zirconate-titanate (PZT), and zinc oxide (ZnO), has brought a turnaround for their development, replacing the conventional brittle ceramics and quartz. Flexible P(VDF-TrFE) is the most ideal piezoelectric material owing to its favorable chemical inertia, simple manufacturing, and large piezoelectric coefficient.

Persano et al. [44] developed a high-performance flexible piezoelectric device based on aligned arrays of P(VDF-TrFE) nanofibers by electrospinning method. The device possessed fast response and high sensitivity, and could detect pressure less than 0.1 Pa, and thus has a great potential in wearable devices. Akiyama et al. [43] demonstrated a high-performance piezoelectric sensor with AlN deposited on polyethylene terephthalate (PET) films. The piezoelectric sensor is composed of two platinum (Pt) thin films, an AlN thin film and a PET film, transferring imposed pressure into electric charges. The SEM image in Figure 4c shows a cross-section micrograph of the AlN layer, which indicated that the columnar nanograins within the AlN layer are perpendicular to the PET film’s surface. In addition, they explored the piezoelectric responses of the sensor by imposing pressure from 0 to 8 MPa at room temperature. Figure 4d shows the dependence of the electric charge on pressure and the dependence of the sensor response on frequency, respectively. They conducted a test for measuring the pulse waves by the fingers and wrist to evaluate the response and sensitivity of the sensor. As shown in Figure 4e, Chen et al. [45] proposed a novel static measurement pressure sensor with nanowire/graphene heterostructure. Compared with traditional piezoelectric NW or graphene pressure sensor, the proposed sensor can measure static pressure with sensitivity up to 9.4 × 10^3^ kPa^−1^ and the response time can reach 57 ms. This pressure sensor shows a great potential for applications in E-skin and wearable devices.

### 2.2. Materials for Flexible Sensors

To a large extent, the properties of material determine the performance of devices, such as arbitrary folding and bending without affecting other performances of devices, which depend on the characteristics of the materials. Materials for flexible devices should not only have an appropriate elastic modulus, but also high mechanical and electrical performances required in large-area arrays. Herein, we elaborate on the materials including flexible substrates, conductors, semiconductors, and dielectrics employed in flexible sensors [44]. 

#### 2.2.1. Flexible Substrates

There are various flexible substrates used for sensors applied in various fields. Polyimide (PI), which has high mechanical and dialectical properties, as well as high chemical stability and thermostability, is widely used in flexible substrates. Its application scope is limited by its characteristic of yellow transparency, however, its cost is high. PET film is widely used because of its low price and comprehensive performance, such as remarkable transparency, toughness, tensile strength, and thermal resistance. Stretchability is drawing the interest of researchers for applications in E-skin and wearable devices. PDMS is regarded as the best choice for stretchable substrates owing to its excellent flexibility and high thermal and dielectric properties [3]. Recently, researchers are devoted to attempting integration of stretchability and high sensitivity of sensors for high flexibility.

#### 2.2.2. Conductors

Recently, conductive materials with high mechanical properties have attracted attention from researchers in highly flexible and stretchable applications [46]. Generally, nanoscale materials, such as nanoparticles, NWs, and nanotubes, are considered as core materials for flexible sensor manufacturing on account of their remarkable electrical conductivity. Metal nanoparticles, including Au, Ag, Al, and Cu, are considered as the best candidates in an integrated circuit. Moreover, carbon nanoparticles with high electrical conductivity and low cost are most widely used in flexible devices, such as graphene and carbon black. NWs are typical one-dimensional materials with a high aspect ratio of more than 1000, and are considered as conductive materials for applications in OLEDs and wearable sensors [47]. CNT is a representative material of one-dimensional materials.

## 3. Promising Applications of Flexible Pressure Sensors

Studies on flexible pressure sensing technology have made great progress in the last decade, benefitting from their excellent mechanical and electronic performances, and they have great potential for practical applications. Flexible pressure sensing technology boosts and drives the development of other sensing technologies, such as E-skin tactile sensors and wearable pressure sensors, which play a crucial role in the fields of medical diagnosis, physical health detection, and artificial intelligence, among others.

**Figure 5:**
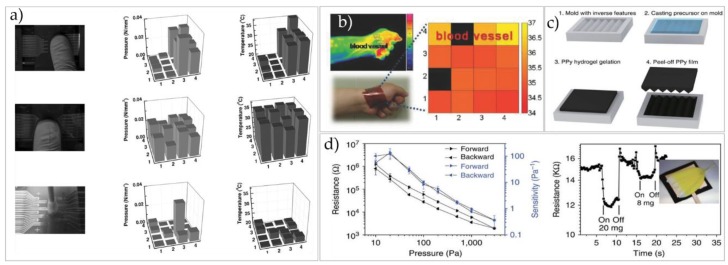
(**a**) The image of an electronic artificial skin. Reproduced with permission [49]. Copyright 2013, Journal of Micromechanics & Microengineering. (**b**) Sensor arrays based on P(VDF-TrFE) microstructure could be applicable for accurate detection of the human body temperature and heartbeat frequency. Reproduced with permission [50]. Copyright 2015, Scientific Reports. (**c**) Fabrication and resistive pressure response of micropatterned elastic microstructured conducting polymer (EMCP) films. (**d**) Resistance response and pressure sensitivity of the EMCP pressure. Reproduced with permission [51]. Copyright 2014, Nature Communication.

However, there are still some difficulties when introducing temperature sensing into practical applications, such as new sensitive materials, especially its matrix arrangement design. The integration of thermal and pressure sensors is firstly proposed by Someya and co-workers [47,48]. They developed an integrated thermal and pressure sensor with great flexibility. For the fabricated large-area arrays based on organic field effect transistors (OFETs), all of their components are made of soft materials except electrodes. Later, Tien and co-workers [49] demonstrated a bimodal flexible sensor array that simultaneously detects pressure and temperature using nanocomposite as dielectric material, which solved the strain problem of the device during operating, as shown in Figure 5a. In order to eliminate or reduce the mutual interference between pressure sensor and temperature sensor to accurately detect the pressure and temperature, many researchers have designed flexible electronic devices on 3D surfaces to form a certain height difference.

### 3.1. Electronic Skin (E-skin) Flexible Tactile Sensors

E-skin flexible tactile sensors have been widely applied to sense the tactician and thermoception of human skin. E-skin can be attached to the surface of a human body or a robot as a garment, and can be processed into various shapes to imitate the sensory function of human skin because of its features of light and softness, and then to achieve intelligence for robots and physiological status detection.

#### 3.1.1. Developments of E-skin Flexible Tactile Sensors

The explorations and studies on E-skin tactile sensors began in the 1970s. Until the 1990s, tactile sensing technology has been developed in directions of flexibility, extensibility, light weight, and multifunctions, especially in recent years. In early times, most tactile sensors were microsensors using silicon as the main material [52], which is realized by adopting MEMS technology. The requirements of flexibility and extensibility cannot be met, however, which stimulated researchers to explore new materials. Kim et al. [50] introduced P(VDF-TrFE) as dielectric to study the applicability of sensor arrays in biomonitoring, such as using sensors to monitor body temperature distribution, as shown in Figure 5b. Lee et al. [53] introduced a ZnO-nanorod/PVDF composite to simultaneously measure temperature and pressure in real time, which could detect temperature ranging from 20 to 120 °C, and a small pressure of 10 Pa. This kind of sensor could be applicable for accurate detection of the human body temperature and heartbeat frequency.

Wang et al. [54,55] proposed a new “sandwich” pressure sensor array based on excellent conductivity and nanometer size effect of silver NWs. The E-skin tactile sensor not only has high flexibility, extensibility, and stability, but also could achieve a wide range of precise measurements, which lay a solid foundation in achieving highly soft elastic E-skin and artificial intelligent robots. Yogeswaran and co-workers [56] fabricated a resistance E-skin flexible elastic tactile sensor for measurement of minimum pressure of 500 Pa, widely used in artificial robots and medical prosthesis, among others. Ding et al. [57] designed a new-type skin liked arrayed tactile sensor based on conductive rubber with a frame structure of two layers of nodes arranged in an intersecting manner. The size of the 3D force on the surface of the flexible sensor could be obtained through the resistance of the conductive rubber under the external force. A series of simulation results show that this flexible tactile sensor array has high resolution and precision in measuring 3D force.

#### 3.1.2. The High Sensitivity of E-skin Tactile Sensors

It is highly important to enhance and develop performance evaluation indexes of sensors, such as resolution ratio, stability, repeatability, and especially the sensitivity of sensors. At present, developing tactile sensors with high resolution, high sensitivity, and rapid response for various applications is the key research goal. In this section, we present a brief introduction of high performances of E-skin tactile sensors. 

Sensitivity is one of the most important parameters of the pressure sensor, and determines the measurement accuracy and validity of the sensor [10]. Sensitivity could be defined as the value of the change in the output result of the sensor compared with the change in the input condition. Lou and co-workers [58] developed a flexible E-skin tactile sensor with high sensitivity and fast response based on graphene nanosheets (GNs), which could detect a slight pressure of 1.2 Pa. Lai fabricated a flexible capacitive E-skin pressure tactile sensor [59], which employed PDMS film as a dielectric layer, and its top and bottom electrodes are Ag NWs. The minimum pressure that could be detected is 0.6 Pa, and the sensor possesses high sensitivity and a fast response time of 20 ms. Pan et al. [51] developed a flexible E-skin pressure tactile sensor with pressure resistance by improving the shape of the microstructure. They found that the sensor could monitor a low pressure below 1 Pa, and its high sensitivity still existed in high pressure areas, as shown in Figure 5c,d. Cao et al. [60] investigated the sensitivity of the flexible tactile sensor based on the force sensitive conductive rubber and discussed the effect of filler amount on sensitivity of composites. Through the discussion on the mechanism of materials, the force sensitivity of material is high when materials move from high resistance to low resistance.

**Figure 6:**
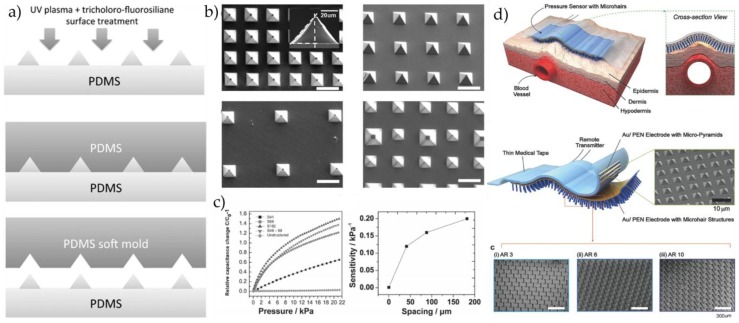
(**a**) Schematic process of the soft PDMS molds. (**b**) Scanning electron microscope (SEM) images of different spatial arrangements of pyramidal microstructured PDMS. Spacing of 41 µm, 88 µm, 182 µm, and interspersed design of two pyramidal structures with different base areas (scale bar: 100 µm). (**c**) Capacitance changes with increasing pressure for different spatial configurations of microstructured PDMS. Reproduced with permission [61]. Copyright 2015, Advanced Functional Materials. (**d**) High skin conforming and pulse-detectable pressure sensor using microhair structures. Reproduced with permission [62]. Copyright 2015, Advanced Materials.

Bao et al. [61] discussed the influence of sensor microstructure on its sensitivity using PDMS as dielectric layer and designed four-pyramid microstructures with different gaps by a soft lithographic molding technique. The preparation process and schematic diagram of dielectric layer are shown in Figure 6a,b. The experimental data showed the following results: (i) with the increase of external pressure, the capacitance increases, and different spatial arrangements of microstructured PDMS varies in trends; (ii) with the increase of microstructure spacing, the sensor sensitivity gradually increases. The sensitivity of the device is up to 0.2 kPa^−1^ when the spacing distance approaches 200 μm, which could applied in various applications, such as capillary pulse detecting or blood pulse monitoring, or could be prepared as the next generation pressure touchpads, as shown in Figure 6c. Bao and co-workers [62] made a new breakthrough in signal-to-noise ratio of a sensor, using medical tape as the base material of the upper plate, and the surface of the plate under the sensor is biomimetic microstructure synthetic cilia. The cilia on the surface of the plate increases the signal-to-noise ratio of the pulse signal to 12 times that of the original (without microstructure), greatly improving the resolution of the device, as shown in Figure 6d.

#### 3.1.3. Challenges and Trends of E-skin Tactile Sensors

E-skin tactile sensing technology has obtained a series of achievements after its recent development in medical services and intellectual industries fields [25]. Improving the environmental adaptability and establishing a new human–machine integration mode through multi-sensor sensing intelligence is the demand of the current development of science and technology [62,63,64].

There are still many difficulties need to overcome. On the one hand, optimizing bionic skin tactile sensor to achieve high tensile rate requires both high flexibility and elasticity, which requires a high level of materials, structure designs, and preparation methods, so the high cost of fabricating tactile sensors limits the mass production of E-skin sensors. For materials, the active materials favored by researchers are carbonaceous materials, including carbon black, CNTs, and graphene, as well as NWs and organic polymers. Carbon based materials are the preferred materials owing to their advantages of light weight, high electrical conductivity, relatively high Young’s modulus, and low cost. Higher requirements for the structural design of E-skin tactile sensors in different fields, and the rise of multi-layer reticular array structure and single-layer array structure [60], micromachining process, and 3D printing technology have created new possibilities for further development of E-skin tactile sensors. Recently, Christ et al. [65] fabricated a sensor using composite thermoplastic polyurethane (TPU)/multi-walled CNTs (MWCNTs) using 3D printing technology and studied its piezoresistive properties. The results showed that the pressure coefficient is as high as 176 at strain up to 100% and maintains a highly repetitive resistance strain response, which is highly promising for applications in wearable devices.

On the other hand, how to achieve extensive coverage and solve the problem of signal crosstalk between adjacent sensor units of sensor array is particularly important. New types of 3D or multi-dimensional flexible tactile sensors are designed with different structures to avoid the influence. Vatani et al. [31] developed an E-skin multi-layer flexible tactile sensor based on a polymer nanocomposite, by adopting direct-print (DP) technology and layer–layer soft forming technology, to provide a new direction for the development of E-skin tactile sensors.

At present, E-skin tactile sensors have achieved flexibility, elasticity, sensitivity, and a wide range by adopting new flexible materials/structures, various sensor array structures, and new manufacturing techniques. Their performance will be challenged with human skin in many aspects, and their research results have been gradually applied in some fields of human production and life, medical treatment, and other fields. Recently, Gao et al. [66] developed a low-cost, sandpaper-type wearable pressure sensor using sandpaper with different sand counts to adjust the sensitivity of the sand by utilizing the fine peaks of sandpaper surface. The sensor has a sensitivity up to 0.2 kPa^−1^, and has a wide pressure test range of 5 Pa to 50 kPa and a fast response time of 0.19 s. However, there is still a large difference between the characteristics of E-skin flexible tactile sensor and comprehensive perceptions of human skin. In addition, most of the existing tactile sensors have single function, mainly focusing on pressure measurement, and only a few of them have the function of simultaneously detecting pressure, temperature, and other parameters, such as humidity and roughness [67]. Therefore, to develop a new type of multi-functional E-skin tactile sensors, with high flexibility, elasticity, and sensitivity, approaching human skin performance is an important research direction in the future.

### 3.2. Wearable Flexible Sensors

Wearable devices have been studied for more than 40 years, and they are widely used in the fields of aerospace, military communication, traffic monitoring, and medical health care detection. In the aerospace field, a new type of infrared temperature sensor with high precision and high durability has a practical application, which could detect the temperature of all parts of the aircraft in operation in extremely bad weather. Infrared photoelectric sensors are widely used in the detection of road sections to prevent accidents, and have been installed in cars to detect road conditions. In recent years, medical and health care problems have aroused wide concerns and interests from the public. Physiological measurements include heartbeat, respiratory rate, blood pressure, blood oxygen saturation, and muscle activity. Highly sensitive pressure sensors have been utilized to collect and analyze fundamental health information of pulse, respiratory rate, and blood pressure to monitor people’s physical health [9,10,11,12,13,14,15,16,17,18,19,20,21,22,23]. Pressure sensors with high flexibility, high sensitivity, light weight, and good workability are urgently needed for wearable health monitoring devices [30].

#### 3.2.1. Development of Wearable Flexible Sensors

With the continuous progress in information technology, the need for developing high- performance flexible sensors, especially wearable pressure sensors, directly attached to the surface of skin to obtain blood pressure, heartbeat, pulse, and a series of health information, which are collected in intelligent equipment for monitoring, is increasing [8,66,67]. At present, the wearable devices represented by the watch, bracelet, running shoes, and belts for movement count, as well as environmental and medical health detection, have dominated the market and will maintain a high growth rate in the next few years according to the data from market research.

Jung et al. [68] fabricated a flexible and biocompatible dry electrode delivering great performance with wearable electrocardiograph (ECG) devices by applying a carbon nanotube–polydimethylsiloxane (CNT–PDMS) composite. In addition, they found that the ECG signal did not change apparently after several days of wearing, revealing that this type of sensor could be applied in long-term wearable healthcare devices. It is necessary to explore flexible pressure sensors to promote the development of wearable pressure devices towards practical applications. Guo and co-workers [69] adopted a novel method for fabricating a wearable sensor vest to implement long-term detection for guiding the treatment and prevention of diseases. A conductive textile fabric is utilized in the sensor vest, which is made of ordinary textile fabrics with a layer of conductive layer formed by immersion plating and solidification with precious metal solution, such as a measuring electrode, and is then sutured on elastic vest. This design is simple, portable, and suitable for daily self-testing. Gao et al. [70] presented a microfluidic tactile diaphragm pressure sensor based on embedded Galinstan micropores (70 m × 70 m) that can be detected by utilizing the tangential and radial strain fields of the embedded equivalent Wheatstone bridge. The pressure range is 50–100 Pa, the response time is 90 ms, and the sensitivity is up to 0.0835 kPa^−1^. In addition, a PDMS smart glove with a microfluidic diaphragm pressure sensor with multiple embedded Galinstan microwells is demonstrated to reflect the touch of a human hand when touching an object.

Wearable flexible sensors exist in the fields of human–computer interaction system and health care monitoring owing to their distinct performances, such as high flexibility, high sensitivity, and high resolution. In order to be applied in various irregular surfaces, there are higher requirements for intelligent materials. Achieving high extensibility and sensitivity, apart from flexibility, needs to be explored. Many achievements in the study of flexible materials are reported. Applying graphene with high electrical conductivity, covered with polyurethane sponges, which are used through several adsorption reduction processes to obtain a flexible strain sensor with low cost, high tensile strength, and high sensitivity, is a development direction in facilitating the progress of sensors technologies [71]. This kind of sensor adopts an electrochemical method, which greatly improves the sensitivity of the device to a certain extent. In addition, wide strain range, fast response, and high repeatability are also prominent through experiments of human finger bending activity monitoring and facial muscle stretching monitoring, showing its great application value in the field of flexible wearable devices.

#### 3.2.2. Wearable Sensors Classification

Different types of sensors are required in wearable devices for achieving sensing functions because of different objects and application scenarios, which can be divided into three classes: motion sensors, biological sensors, and environmental sensors.

Motion sensors are widely used in wearable motion monitoring, navigation, and man–machine interaction, which can actually detect motion-related sensor information, such as posture and position, among others. The common motion sensors include gyroscope, accelerometer, and geomagnetic sensors. Biosensors are composed of biological materials and receptors with exclusive function, acting as an advanced detection method in biotechnology. Biosensor technology is evolving rapidly, such as the recently developed factory-calibrated glucose sensor, which does not require calibration by user even during wear and tear, and can continuously manage diabetes with a blood glucose monitor [72]. Biosensors are widely defined, and are capable of detecting vital signs and disease monitoring and control, such as blood pressure sensors [73], ECG sensors [74], and temperature sensors [75]. Environmental sensors include temperature and humidity sensors, ultraviolet (UV) sensors [76], soil acidity or alkalinity sensors, and light sensors, among others. Based on these sensors, they can be used not only to measure relevant indicators in the environment and to carry out weather forecasts and health warnings, but also to explore the influence of environmental factors on experimental samples in scientific research.

#### 3.2.3. Challenges and Trends of Wearable Sensors

Internet-based medical and health data systems are growing rapidly. Precision medicine is an emerging medical field that focuses on making accurate diagnosis, and wearable sensors will provide a possibility for the development of this field. Doctors can develop effective health care plans in time based on data analysis of patients’ routine monitoring and clinical research. To obtain these data accurately, continuous and repeated measurements will be conducted by wearable devices, which are implemented for sports and sleep monitoring; however, they fall short of all indicators for precision medicine. Combining precision medicine with wearable sensors and big data, and applying the big data to the construction of medical platform could allow one to effectively analyze large measurement data.

Initially, wearable sensors were mainly motion sensors, such as acceleration sensors for recording movement information. Then, pressure sensors, photoelectric sensors, and temperature sensors were developed, which were integrated into wearable devices for health monitoring in blood sugar, blood pressure, and heart rate. These sensors are mainly utilized to measure physical quantities, but there are some difficulties to monitor chemical quantities, such as measurement of particles content. Furthermore, available working hours affected market acceptance, so reducing power consumption should be considered for further development. On account of large amounts of data involving personal habits and privacy stored in wearable sensors, it is necessary to establish a platform to supervise data security for the healthy and stable development of wearable sensors. Apple Inc. has developed a software framework based on a smartphone app and wearable devices for solving data reliability, security, and confidentiality problems, among others, which promoted wearable devices with higher effectivity in health management and medical treatment [30].

Wearable products such as smartwatches and bracelets, which are currently in rapid growth, are indicative of the increasing acceptance of wearable products. In the future, the research on implantable wearable devices and thinner skin sensors will become a new breakthrough point, and wearable sensors are moving toward higher performance and smaller size.

## 4. Conclusions and Outlook

In this review, we highlight the progress of flexible pressure sensors and their applications in E-skin and wearable flexible devices. Recently, researchers achieved great progress in functional materials and structure design of 3D sensor arrays to obtain reduced mutual interference in signals. Tremendous advances in the development of flexible pressure sensors have made them widely used in practical applications. E-skins with significantly improved performance dominate the field of artificial intelligence to promote the development of intelligent technologies. By virtue of the introduction of processing chips with high performance and low power consumption, and the development of various sensors, wearable intelligent devices have gone from concept to commercialization, which in turn promote the development of sensors by integrating sensors into wearable devices.

However, some challenges still remain for practical applications of flexible sensors. For example, new materials and innovative conductive mechanisms should be further explored to achieve a wider area of pressure measurement in the field of medical implant services and health monitoring, as well as other potential fields. Moreover, the microfabrication and production of devices should keep up with the demand and development of sensors. In addition, different types of pressure sensors are required in different fields, which are highly dependent on the application field. The current level of performance of sensors is below the requirements in practical applications. In terms of sensors applied in real life, integrating pressure, temperature, and other perceptions into a combined sensor is taking over the market of sensors. Future sensor technology will precisely respond to variations in the external environment, which presents a vital part in the fields of intelligent robots, and medical and human activity for health monitoring.

## Figures and Tables

**Table 1:** A summary of the reported performance of flexible pressure sensors. PFA—perfluoroalkoxy alkane; PVDF—polyvinylidene difluoride; CNT—carbon nanotube; VACNT—vertically aligned CNT; PDMS—polydimethylsiloxane; OFET—organic field effect transistor.

Active Materials	Perception Mechanism	Sensitivity	Minimum Detection	Maximum Detection	Ref.
PFA	Piezoelectricity	15 V kPa^−1^	-	2.5 kPa	[19]
PVDF	Piezoelectricity	-	1 kPa	30 kPa	[20]
ZnO nanorod	Piezoelectricity	-	3.5 kPa	31.5 kPa	[21]
CNTs/PDMS interlocked microdome	Piezoresistivity	15.1 kPa^−1^	0.2 Pa	59 kPa	[22]
ACNT/G/PDMS	Piezoresistivity	19.8 kPa^−1^	0.6 Pa	0.3 kPa	[23]
VACNT/PDMS	Piezoresistivity	0.3 kPa^−1^	2 Pa	10 kPa	[24]
Graphene-paper	Capacitance	17.2 kPa^−1^	2 kPa	20 kPa	[25]
PDMS microstructure OFET	Capacitance	0.55 kPa^−1^	3 Pa	20 kPa	[26]

**Table 2:** Comparison of advantages and disadvantages of conductive fillers.

System	Classification	Advantages	Disadvantages
Metal System	-	Excellent electrical conductivity Great chemical stability Preservative	Easy oxidization Instability in conductivity High hardness Difficult forming
Carbon System	Graphene	Excellent electricity Low cost	Instability in conductivity Difficult to form chain aggregates
	Carbon Black	Great and stable electricity Easy for preparation Low cost Rich in variety	Easy to reunite
	Carbon Nanotubes	Excellent electricity Great surface area	Complex for preparation High cost
